# Computer-aided drug design of *Azadirachta indica* compounds against nervous necrosis virus by targeting grouper heat shock cognate protein 70 (GHSC70): quantum mechanics calculations and molecular dynamic simulation approaches

**DOI:** 10.5808/gi.21063

**Published:** 2022-09-06

**Authors:** Sk Injamamul Islam, Saloa Saloa, Sarower Mahfuj, Md Jakiul Islam, Moslema Jahan Mou

**Affiliations:** 1 Department of Fisheries and Marine Bioscience, Faculty of Biological Science, Jashore University of Science and Technology, Jashore 7408, Bangladesh; 2 Department of Environmental Science and Technology, Faculty of Applied Science and Technology, Jashore University of Science and Technology, Jashore 7408, Bangladesh; 3 Faculty of Fisheries, Sylhet Agricultural University, Sylhet 3100, Bangladesh; 4 Department of Genetic Engineering and Biotechnology, Faculty of Life and Earth Science, University of Rajshahi, Rajshahi 6205, Bangladesh

**Keywords:** *Azadirachta indica*, dynamic simulation, grouper heat shock cognate protein 70, molecular docking and ADMET, nervous necrosis virus

## Abstract

Nervous necrosis virus (NNV) is a deadly infectious disease that affects several fish species. It has been found that the NNV utilizes grouper heat shock cognate protein 70 (GHSC70) to enter the host cell. Thus, blocking the virus entry by targeting the responsible protein can protect the fishes from disease. The main objective of the study was to evaluate the inhibitory potentiality of 70 compounds of *Azadirachta indica* (Neem plant) which has been reported to show potential antiviral activity against various pathogens, but activity against the NNV has not yet been reported. The binding affinity of 70 compounds was calculated against the GHSC70 with the docking and molecular dynamics (MD) simulation approaches. Both the docking and MD methods predict 4 (PubChem CID: 14492795, 10134, 5280863, and 11119228) inhibitory compounds that bind strongly with the GHSC70 protein with a binding affinity of ‒9.7, ‒9.5, ‒9.1, and ‒9.0 kcal/mol, respectively. Also, the ADMET (absorption, distribution, metabolism, excretion, and toxicity) properties of the compounds confirmed the drug-likeness properties. As a result of the investigation, it may be inferred that Neem plant compounds may act as significant inhibitors of viral entry into the host cell. More *in-vitro* testing is needed to establish their effectiveness.

## Introduction

Viral nervous necrosis (VNN) disease, also called viral encephalopathy and rectinopathy, viral vacuolating, encephalopathy and rectinopathy or piscine neuropathy. Nervous necrosis virus (NNV) of the genus Betanodavirus (25‒30 nm) is the causative agent of VNN and it consists of four genotypes: among them, the red spotted grouper nervous necrosis virus genotype shows comprehensive host range [1]. All growth stages of fish are affected heavily by NNV but mass mortalities were reported in marine fin fishes, especially among larvae less than 20 days old [2,3]. There are at least five orders of fish that may be affected by this virus which means it can infect 16 different families of fish. Since the virus is spread by water, it can affect healthy and sick fish in the same area [3] and when this virus examined under light microscopy the target organ of NNV, is the spinal cord mainly the central nervous system of the infected fish and marked vacuolations in the eye retina and brain of fish [4]. Japan is probably the first country where nodavirus infection was detected in Japanese parrotfish (*Oplegnathus fasciatus*) [5] and afterwards infection from nodavirus has recorded in about 40 species till date, causes mass mortality and resulted tremendous economic damages globally in last two decades.

Two-single-stranded positive-sense RNAs are the main compound for NNV genome structure [6]. RNA-1 is responsible for RNA-dependent RNA polymerase encode and viral capsid protein encoded by RNA-2 [7]. Additionally, B-1 and B-2 proteins which are the function of a small nonstructural protein are encoded by a sub genome of RNA-1 named RNA-3 of betanodaviruses. B-1 and B-2 proteins are encoded by a sub genome of RNA-1 named RNA-3 [8]. The B-1 protein shows early stage of infection, representing an anti-necrotic cell demise function by reducing mitochondrial membrane protein loss and thus, enhancing cell viability [9]. RNAi-mediated cleavage by a host, on the other hand, is inhibited by the B-2 protein, which functions as a binder between intermediate double-stranded RNA and NNV [10]. The only structural protein of the virion is the NNV coat protein and has been tested to fix the host range [11]. Monoclonal antibodies specific for NNV with high neutralizing titers have been produced [12], indicating that NNV-specific receptors are present in host cells. Virus infection by NNV occurs through receptor-mediated endocytosis and macro pinocytosis. Since susceptible SSN-1 cells (derived from striped snakehead) contain sialic acid residues that are compatible with virus, these are the sites where the virus attaches [13].

All the knowledge and information related to NNV invasion into the host cell remains limited till now. Grouper heat shock cognate protein 70 (GHSC70) and grouper voltage-dependent an ion selective channel protein 2 were examined as NNV receptor protein candidates utilizing the grouper fin cell line GF-1 and purified NNV capsid protein in a viral over lay protein binding assay. The GHSC70 protein acts as a NNV receptor or coreceptor in GF-1 cells, most likely acting as a receptor [14].

Medicinal plants can play a critical role in the treatment of a variety of ailments, particularly in areas where resources are scarce. Traditional remedies are mostly advocated given the abundance of these plants all over the world [15]. Before anything, traditional drugs have less detrimental consequences than modern drugs, which is one of the main reasons why essential chemicals are extracted and produced from plants [16]. *Azadirachta indica* is a typical medicinal plant whose importance has risen steadily in recent years around the world. It contains a large number of biologically active compounds with a variety of structures. Well over 140 effective chemical compounds have been reported and extracted from various components of this plant, which include leaves, flowers, seeds, roots, fruits, and bark have been used traditionally as a treatment for a variety of diseases, as shown in a research. Anti-inflammatory, immune-modulator, anti-mutagenic, anti-carcinogenic, anti-oxidant, and anti-viral medicines have all been found in these potent molecules [17].

*A. indica* components are divided into two categories: non-isoprenoids and isoprenoids. Proteins, sulphurous molecules, carbohydrates, and polyphenolics such as dihydrochalcone, flavonoids, coumarin, and aliphatic molecules are all examples of non-isoprenoid. Azadirone, protomeliacins, limonoids, and some derivatives including nimbin, vilasinin, salanin, and azadirachtin are among the di-terpenoids and tri-terpenoids used to make isoprenoids [15].

To introduce effective medicines in a conventional or standard manner can take a long time, be expensive, and require a significant amount of effort [18]. For example, high-throughput screening (HTS) is a technique that integrates multiple-well microplate with automated processing to improve drug development by assaying a large number of putative drug-like molecules [19]. Additionally, HTS should have abundant resources, as processing a particular HTS program is expensive and involves the use of robotic devices [20]. On the contrary, computer-aided drug design, also known as *in silico* drug design, is a relatively new technology for screening a large database of compounds using a high-throughput approach [21]. The *in silico* virtual screening approach aids in the discovery of novel medicines by generating hits for lead compounds in a shorter period and at a cheaper cost [22]. As a result, improved *in silico* drug design reduces the time required to develop, design, and optimize a novel drug. The virtual screening approach has been used for decades to find the best lead compounds with various structural properties for use with a given biological target [23]. Furthermore, computer-aided drug design has been used to find a wide variety of interesting drug applications and hits utilizing virtual screening, molecular docking, and dynamics simulation techniques [24]. In light of the above-mentioned *A. indica* drugs, the goal of this study is to use molecular docking to screen active compounds of *A. indica* against the GHSC70 and investigate their interaction pattern. As a result, the goal of this work was to combine virtual screening, molecular docking, and ADMET (absorption, distribution, metabolism, excretion, and toxicity) features strategies to screen potential natural anti-fish drugs.

## Methods

### Retrieving the sequence

The UniProtKb database (https://www.uniprot.org/) was used to retrieve the amino acid (aa) sequence of the GHSC70 protein (UniProtKb ID: A0A096VJY) found in NNV and downloaded in FASTA format.

### Assessment of secondary structure

The secondary structural elements of the protein GHSC70 were predicted through the SOPMA tool [25] using the default parameters (window width of 17, number of states of 4, and similarity threshold of 8).

### Prediction, refinement, and validation of three-dimensional structures

The three-dimensional structure of the target protein was predicted using the Raptorx server (http://raptorx.uchicago.edu/) [26]. The protein 3D structure was refined by GalaxyWeb server (https://galaxy.seoklab.org). The structure validity is a crucial stage in homology modeling, which is based on experimentally validated the structure of 3D proteins. A 3D model of the target protein is developed based on a sequence alignment between the target protein and the template structure [27]. The protein 3D structure was refined by GalaxyWeb server. The structure's validity is a crucial stage in homology modeling, which is based on experimentally validated the structure of 3D proteins. The proposed GHSC70 protein model was uploaded to ProSA-web for basic confirmation [28]. The server foresaw the overall character of the model, which is represented by the z-score. If the expected model's z-scores are outside the scale of the property for local proteins, it indicates that the structure is erroneous [28]. To determine the overall quality of the suggested drug, a Ramachandran plot analysis was performed using the Ramachandran Plot Server (https://zlab.umassmed.edu/bu/rama/) [29].

### Preparation of protein

The protein's 3D structure was modelled and developed using the following criteria: water, metal ions, and cofactors were removed, polar hydrogen atoms were introduced, nonpolar hydrogen was combined, and gasteiger charges were calculated using AutoDockTools [30].

### Retrieval and preparation of compounds

Phytochemicals derived from naturally occurring medicinal plants cover a wide range of chemical spaces that can be used in drug development and discovery. IMPPAT stands for Indian Medicinal Plants, Phytochemistry, and Therapeutics, a manually curated database of over 1,742 Indian medicinal plants and over 9,500 phytochemical compounds that uses cheminformatic methodologies to improve natural product-based drug discovery [31]. Because of virtual screening, the phytochemical of the neem plant (*A. indica*) has been discovered and obtained from the database. The compounds found from the database were created by assigning accurate AutoDock 4 atom types, merging nonpolar hydrogens, detecting aromatic carbons, and establishing a ‘torsion tree. It has been discovered that the AD4 atom type is the same as the compound's elements for the majority of atoms.

### Molecular docking and receptor grid generation

The PyRx virtual screening tool AutoDock Vina was used to create a protein receptor grid [32]. The molecular docking investigation was carried out using the PyRx virtual screening program AutoDock Vina to find the binding mechanism of the required protein with chosen phytochemicals. PyRx is an open-source virtual screening application that can screen libraries of compounds against a given therapeutic target and is primarily used in Computer-Aided Drug Design (CADD) techniques. PyRx integrates AutoDock 4 and AutoDock Vina as docking wizards with an intuitive user interface, making it a more trustworthy CADD tool. This experiment used PyRx's AutoDock Vina wizard for molecular docking to find the optimum protein and ligand binding poses. For docking objectives, the default configuration parameters of the PyRx virtual screening tools were utilized, and the highest binding energy (kcal/mol) with the negative sign was chosen for further investigation. Subsequently, using the BIOVIA Discovery Studio Visualizer v19.1.0.18287, the binding interaction of the protein–ligands complex was seen.

### Predicted pharmacology

The physicochemical, pharmacokinetics, metabolism, and excretion properties of molecules into urine and feces are all listed in the ADME of a substance [33]. The Swiss-ADME server (http://www.swissadme.ch/) was used to forecast the various pharmacokinetic and pharmacodynamic parameters for the experiments [34]. In the area of drug discovery and development, initial analysis of a compound's toxicity is critical [35]. Toxicology profiles of drug candidates provide information about the hazards to human health and the environment, as well as the safety and toxicity of chemical constituents. Chemical toxicity is now assessed using computer-assisted *in-silico* testing without the need for animal experiments. As a result, the ProTox-II (http://tox.charite.de/protox II) website was used to assess the early-stage toxicity of the chosen medication candidates. With ProTox-II, you can identify compounds that are acutely toxic, hepatotoxic, cytotoxic, carcinogenic, mutagenic, and immunotoxic [36]. Using quantitative structure-activity relationships techniques, the software estimates the toxicity of specified compounds.

### Quantum mechanics‒based calculation

When it comes to determining possible active conformation, binding affinity, and strain discipline within a binding process, there is a requirement for conformation analysis of the ligand to the binding site. In such an instance, structural optimization and lowest energy conformations can be used, which require gas-phase energy and the solution phase. A ligand-protein complex system with metal ions does not lend itself to the conventional molecular mechanics mechanism [37]. Using quantum mechanical calculations, scoring functions have been developed that explain electronic structure and electronic changes, as well as system-specific charges during a system's reaction. A surprising amount of quantum mechanics (QM) based computations are currently based on density functional theory (DFT). As a result, the DFT methods-based QM calculations of three substances were done in this work. Initially, the bond lengths, bond angles, and dihedral angles for potential compounds were optimized, then the DFT of the compounds has been calculated by using the ORCA quantum chemistry program package (version 4.1.1) [38,39]. The dispersion correction energy term D3 was used with Becke's three parameters (B3LYP) and Lee-Yang-Parr functionals (B3LYP-D3) to calculate DFT. The conventional combination of functionalities B3LYP-D3 was chosen for this investigation because it does not directly affect the wavefunction or any other molecular characteristic, and 6-31G**, also known as 6-31G (d, p), was chosen as a basis set to describe the molecules electronic wave function.

### Frontier molecular orbital HOMO/LUMO calculation

Based on Kenichi Fukui's frontier molecular orbital (1950s FMO) hypothesis, Fukui functions, are the highest energy occupied (HOMO) and lowest energy unoccupied (LUMO) orbitals. FMOs are electron frontier that help determine the energy difference between HOMO and LUMO orbitals. In nature, HOMO is primarily an electron donor (nucleophilic) and LUMO is primarily an electron acceptor (electrophilic), and the interaction between the electron donor and electron acceptor pair can influence other chemical reactivity of a molecule [40]. Electrons from the HOMO jump to the LUMO during the electrophilic-nucleophilic process, resulting in an energy differential between two molecular orbitals. The HOMO-LUMO gap is the difference in energy between two molecular orbitals, that illustrates photochemistry as well as the strength and stability of organic transition metal complexes. To get a better understanding of atom susceptibility to electrophilic and nucleophilic assaults, the HOMO and LUMO energy were calculated by using the Avogadro software and visualize by Avogadro and Chemcraft software [39], and the energy difference between two molecular orbital HOMO-LUMO gaps was calculated from the following Eq. (3).


(1) $$∆E(\mathrm{gap})\;=\;E_{\mathrm{LUMO}}-E_{\mathrm{HOMO}}$$

here, ∆E is the HOMO-LUMO gaps, E_LUMO_ is the lowest energy unoccupied molecular orbital energy, and E_HOMO_ is the highest energy occupied molecular orbital energy.

### Molecular dynamics simulation

The binding stability of the selected candidate compounds to the desired protein to the active site cavity of the protein was assessed using 50 ns molecular dynamic simulations (MDS) [41]. The MDS of the receptor-ligand complex was carried out using the 'Desmond v6.3 Program' in Schrödinger 2020-3 under the Linux framework to assess the receptor-ligand complex's thermodynamic stability [42]. A preset TIP3P water model was utilized to solve the system, with an orthorhombic periodic boundary box shape with a box distance of 10 Å to both sides to maintain a certain volume. Appropriate ions, such as Na^+^ and Cl^‒^, with a salt concentration of 0.15 M, were chosen and inserted randomly in the solvated system to electrically neutralize the state. The system was reduced and relaxed using the default protocol introduced within the Desmond module with OPLS 2005 force field settings after generating the solvated system comprising protein in complex with the ligand [41]. NPT ensembles were kept at 300 K and one atmospheric (1.01325 bar) pressure using the Nose-Hoover temperature coupling and isotropic scaling approach, followed by 150 PS recording intervals with an energy of 1.2. All MDS pictures were taken with Maestro v-12.5. Using the Simulation Interaction Diagram (SID) of Desmond module v6.3, the root-mean-square deviation (RMSD) and root-mean-square fluctuation (RMSF) were utilized to evaluate the stability of the complex structure based on the 150 ns trajectory performance.

## Results

### Sequence retrieval and secondary structure inquiry

**Fig. 1.**
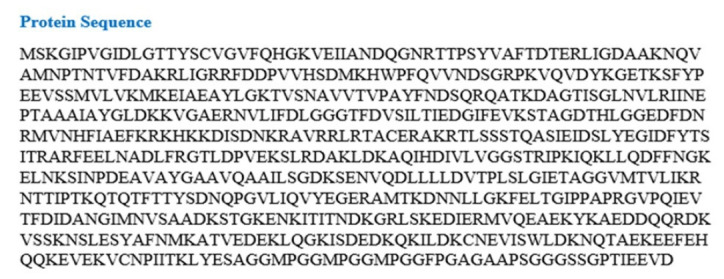
The amino acid (aa) sequence of the protein of nervous necrosis virus.

**Fig. 2.**
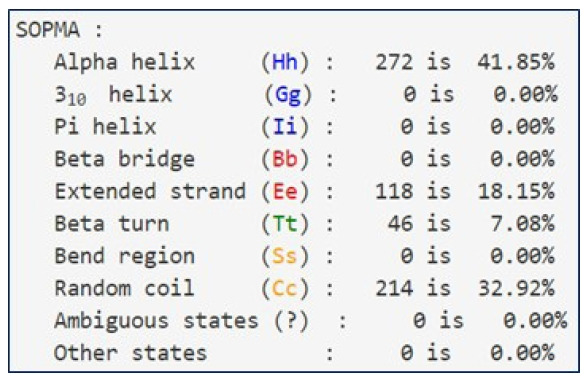
Secondary structural elements predicted by SOPMA server.

The amino acid (aa) sequence of the NNV protein (UniProtKb ID: A0A096VJY) was obtained from the NCBI database. There are 650 amino acids in the protein. Fig. 1 provides additional information on the protein (UniProtKb ID: A0A096VJY). The alpha helix (Hh), extended strand (Ee), beta turn (Tt), and random coil (Cc) of the protein (A0A096VJY) were predicted by the SOPMA software to be 272 (41.85%), 118 (18.15%), 46 (7.08%), and 214 (32.92%) (Fig. 2). Most proteins contain the α-helix, which is a fundamental structural element. Α-helices are formed by hydrogen bonds between the carbonyl oxygen of one peptide bond and the amino acid located three amino acids away. β-strands are also important structural elements of proteins. The protein chains are predominantly linear when β-strands are present. Furthermore, some portions of the protein chain do not form a regular secondary structure or have a consistent hydrogen-bonding pattern. These regions are known as random coils and are found in two locations in proteins: (a) terminal arms and (b) loops.

### Three-dimensional structure prediction, refinement, and validation

**Fig. 3.**
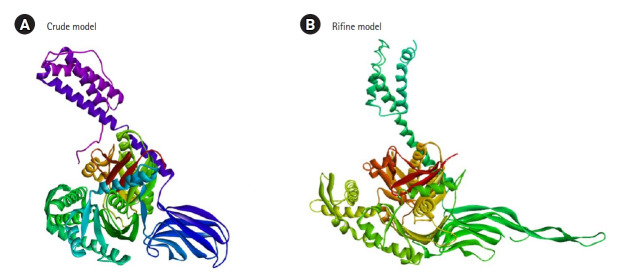
(A) 3D structure of crude model. (B) 3D structure of refine model.

The 3D structure of the protein model predicted using the RaptorX server (http://raptorx.uchicago.edu/). The Galaxy Refine server was used to refine the predicted protein tertiary structure, yielding five refined models and increasing the amount of amino acid residues in the favored location. When compared to the other models, the scores listed above indicate the improved model's caliber. Crude model and refine model 1 (RMSD value 0.409) were chosen and visualized in Pymol (Fig. 3). Ramachandran Plot Server and ProSA-Web online server were used to validate the before and after revised GHSC70 protein model. Ramachandran plot analysis of the before refine structure revealed that 96.649% of the structure was in the favorable zone, as per Ramachandran plot server. After refining, the rampage server produced a better result, with 98.765% of residues in the preferred regions (Table 1). The validation quality and potential faults in a basic tertiary structure model are assessed using the ProSA-web server. Validation of the final GHSC70 protein model reveals a Z-score of ‒11.24 (Table 1, Supplementary Fig. 4).

### Retrieval and preparation of phytochemicals

The Indian natural and medicinal phytochemical compound library (IMPPAT database) was used to find the accessible compounds of the required plant. A list of 70 chemicals was discovered in the database from the Neem (*A. indica*) plant (Supplementary Fig. 5). The phytochemical components found in neem plants were extracted and recorded in a 2D (SDF) file format. During the ligand preparation procedures, the compounds were produced and optimized, then converted to pdbqt file format for further assessment.

### Molecular docking analysis

**Fig. 4.**
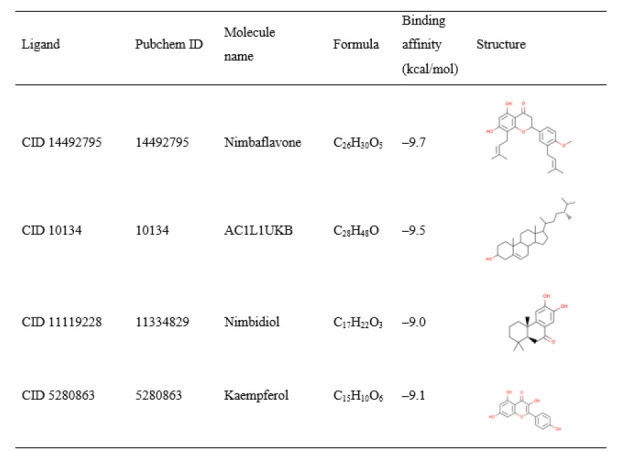
The top 4 compounds' molecular docking score and ligand structure.

A molecular docking study was first conducted to screen and identify the optimal intermolecular interaction among the desired protein and phytochemical substances. PyRx tools AutoDock Vina wizard was used to perform molecular docking between 70 phytochemical compounds and their proteins of choice. The binding affinities discovered during molecular docking of the phytochemical molecule reported in Supplementary Fig. 5. Based on the binding affinity top 4 of 70 phytochemical (total 4) compounds have been chosen (Fig. 4). The docking methods predict 4 (PubChem CID: 14492795, CID: 10134, CID: 5280863, and CID: 11119228) inhibitory compounds that bind strongly with the GHSC70 protein with a binding affinity of ‒9.7, ‒9.5, ‒9.1, and ‒9.0 kcal/mol, respectively (Fig. 4).

### Predictive pharmacology

The ADME characteristics of chemical compounds are crucial in determining a drug's effectiveness. Pharmacokinetics-related failure in clinical stages can be reduced by optimizing ADME characteristics, which is complex and demanding in the drug design and trial process [34]. It has been discovered that assessing ADME at an early stage in the clinical drug development process can lower attrition rates. As a result, the SwissADME online tool was used to conduct an early-stage evaluation of ADME characteristics for four drugs. Focusing on hydrophilic nature, solubility, pharmacokinetics, medicinal chemistry, and drug-likeness characteristics, the server assessed the ADME qualities of four compounds (CID: 14492795, CID: 10134, CID: 5280863, and CID: 11119228). All the compounds have maintained an optimum pharmacokinetics property (Table 2). Toxicity testing is an essential and crucial phase in pharmaceutical development that aids in determining the adverse levels of toxic compounds on people, wildlife, plants, and the surroundings. Traditional toxicity testing of chemicals necessitates the use of an *in vivo* animal model, that is time-consuming, costly, and fraught with ethical issues [36]. As a result, computer-aided *in silico* toxicity measurements of chemical compounds might be regarded beneficial in the drug development phase. The study used the ProTox-II web server to compute the toxicity of the chemical since it is quick, inexpensive, and does not need any ethical concerns. The four compounds (CID: 14492795, CID: 10134, CID: 5280863, and CID: 11119228) selected previously through different screening process have been submitted in the ProTox-II web server that determines the acute toxicity, hepatotoxicity, cytotoxicity, carcinogenicity, and mutagenicity of the compounds listed in Table 3. All the compounds have shown no oral toxicity or organ toxicity effect.

### Geometry optimization

**Fig. 5.**
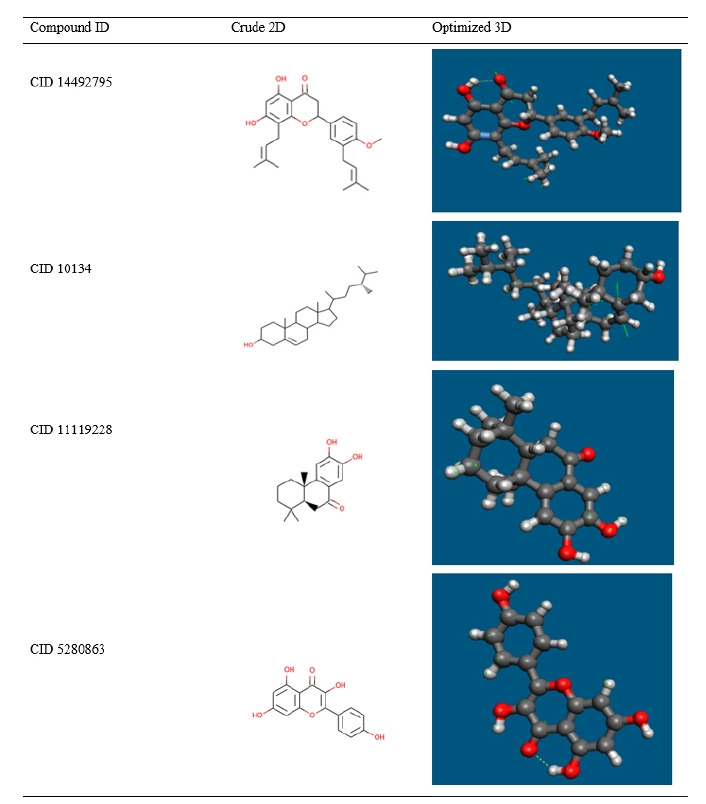
Geometry optimization of selected four compounds.

Most computational biologists, chemists, academicians, and researchers utilize geometry optimization, a quantum chemical approach, to discover the configuration of least energy with the most stable form of a chemical properties. This is a technique for taking crude geometric approximations and perfecting them [42]. Because molecules in the lowest energy state naturally lower their energy by emitting, the geometry with the lowest energy is the most stable. Using the default basis set 6-31G (d,p) in Avogadro, the most optimized molecular shape with the lowest energy value has been established. The 2D structures and 3D optimized geometries of the compounds CID: 14492795, CID: 10134, CID: 5280863 and CID: 11119228 have been plotted in Fig. 5.

### Frontier molecular orbital HOMO/LUMO calculation

**Fig. 6.**
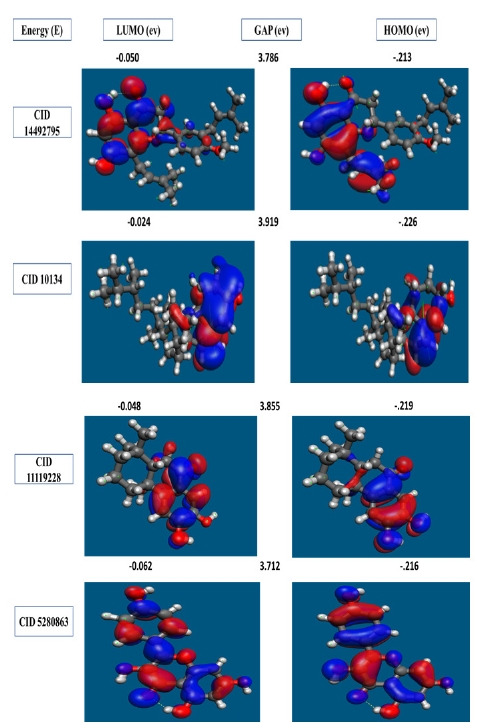
The molecular frontier orbital wave function is shown with negative and positive phases for selected four compounds, representing asymmetric HOMO, LUMO, and HOMO-LUMO gaps.

In organic chemistry, the FMO is increasingly widely utilized to describe the structure and reactivity of molecules. HOMO-LUMO bandgap energy is used in the theory to describe the electrical and optical characteristics of molecules. The energy gap between the two orbitals HOMO and LUMO also helps to determine the sensitivity of atoms toward electrophilic and nucleophilic attacks, chemical kinetic stability, chemical hardness, and softness of a molecule. The electrons in the HOMO orbital are the freest to engage in nucleophilic reactions, whereas the electrons in the LUMO orbital participate in electrophilic reactions. A soft molecule is one that has a low HOMO-LUMO gap energy and a high chemical reactivity while also having a poor kinetic stability. A molecule with a high frontier (HOMO-LUMO) orbital gap should have low chem reactivity or bioactivity and high kinetic stability in this process due to the limited likelihood of attaching an electron to the high-energy LUMO. When compared to a molecule with a low FMO energy gap, molecules with a large FMO energy gap are energetically stable due to low chemical reactivity and high kinetic stability [42]. Therefore, to evaluate the chemical reactivity and kinetic stability of the selected three compounds the HOMO, LUMO, and HOMO-LUMO, gap energy was calculated from Eq. (1) and shown in Fig. 6. The calculated FMO energy band gap values found for the compounds CID: 14492795, CID: 10134, CID: 5280863 and CID: 11119228 was 3.786 eV, 3.919 eV, 3.712 eV, and 3.855 eV, respectively, which was considerably higher, indicating kinetic stability and low chemical reactivity of the molecules.

### Re-docking and interaction

#### Redocking score

The re-docking procedure was used to find potential docking poses in a limited area by utilizing previously acquired protein binding sites. The geometry optimized structure has been docked and the score found for the selected three compounds CID: 14492795, CID: 10134, CID: 5280863, and CID: 11119228 were −10.4 kcal/mol, −9.9 kcal/mol, −9.9 kcal/mol, and −9.5 kcal/mol respectively, which was better than the previously obtained binding score (Fig. 4). As a result, it can be concluded that the QM-based compound optimization was successful for the three compounds chosen.

#### Protein–ligands interaction interpretation

**Fig. 7.**
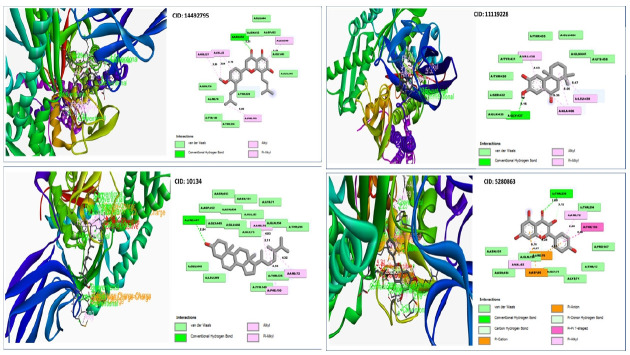
Depicted the interactions between the compounds and grouper heat shock cognate protein 70.

With the desired GHSC70 protein model, the compound CID:10134 produced two Pi-Alkyl interactions with ARG76(4.83) and ARG76 (5.11), where two Alkyl bonds was discovered to form at the positions ARG72 (4.32) and PHE150 (4.56) (Table 4, Fig. 7).

The interaction investigation of the compound CID: 5280863 revealed two Pi-Alkyl bonds at the location of ARG72 (4.46) and VAL82 (5.16) and one Pi-Anion bonds at the residual positions of ARG76 (4.01) and one Pi-Cation at the residual positions of ASP80 (4.47). One conventional hydrogen bond interaction at the position of THR226 (2.89) and one carbon hydrogen bond formed at the position of THR216 (3.12) (Table 4, Fig. 7).

With the target protein, compound CID: 11119228 has been found to create single conventional hydrogen bonds at the positions GLY437 (2.15) and 2 Pi-Alkyl bonds at the positions ALA406 (5.05) and ALA406 (5.35) (Table 4, Fig. 7).

Conventional hydrogen bonds were observed to form exclusively at the TYR15 (2.8579) position of the molecule CID:14492795, where pi– anion and alkyl bonds have been observed at the positions of GLU268 and VAL369, where the distance for the Pi-Alkyl bond was 4.6868 and the distance for Alkyl bonds was 5.4303 as shown in Table 4 and Fig. 7.

### RMSD of protein

The RMSD of the three compounds chosen was used to determine the differences in protein structure when compared to the beginning point. It also aids in determining the protein's equilibration status, which is defined by the flattening of the RMSD curve. The protein frames and the backbone of the reference frame were initially aligned. The RMSD of the system was determined based on the atom selection during the MDS. The complex system with a time frame x should have the RMSD that can be calculated from the following Eq. (2).


(2) $$RMSF_{x}=\sqrt{\frac{1}{N}}\sum_{i=1}^{N}<{\left(r_{i}^{'}\left(t_{x}\right)-\;r_{i}\;\left(t_{ref}\right)\right)}^{2}$$

Here, the RMSDx is the calculation of RMSD for the specific number of frames, *N* is the number of selected atoms; *t_ref_* is the reference or mentioned time, and *r'* is the selected atom in the frame *x* after superimposing on the reference frame, *t_x_* is the recording intervals.

**Fig. 8.**
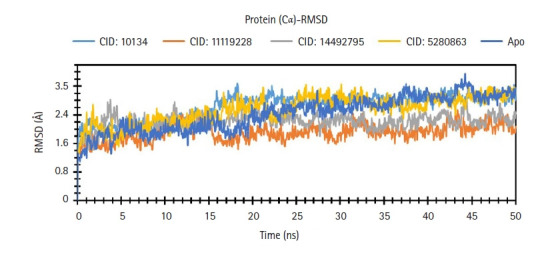
Root-mean-square deviation (RMSD) values retrieved from protein fit lig atoms of the complex structure, viz. CID: 10134 (sky blue), CID: 11119228 (orange), CID: 14492795 (gray), and CID:5280863 (yellow), and grouper heat shock cognate protein 70 backbone or apo (deep blue) for a 50 ns simulation time.

RMSD has been determined for compounds CID: 10134 (sky blue), CID: 11119228 (orange), CID: 14492795 (gray) and CID: 5280863 (yellow), and GHSC70 backbone or Apo (deep blue) based on the selection of the ligand fit protein atom shown in Fig. 8. Except for the compound’s CID:10134 and CID:5280863, the RMSD data revealed that the two compounds were stable, but all compounds are more stable when compared to Apo protein. The average value change of the CID: 11119228 and CID: 14492795 compounds was 0.0‒0.3Ȧ, with the value change for the compound CID:10134 and CID: 5280863 being >3.2Ȧ, which was more over the required range, indicating the protein's substantial conformational shift (Fig. 8).

### RMSF analysis

The RMSF is useful for observing local changes in a protein because it allows you to calculate the average change seen over a large number of atoms, which allows you to estimate the displacement of a single atom in comparison to the reference structure [43]. This is a numerical computation similar to RMSD that may be used to characterize a protein and determine the flexibility and fluctuation of the residues during simulation. The RMSF for residue *i* has been calculated from the following Eq. (3).


(3) $$RMSF_{i}=\sqrt{\frac{1}{T}}\sum_{t=1}^{T}<{\left(r_{i}^{'}\left(\text{t}\right)-\;r_{i}\;\left(t_{ref}\right)\right)}^{2}>$$

where *T* is the overall trajectory time, $r_{i}^{'}\left(\text{t}\right)$ is the residue location, *t_ref_* is the reference time, *r'* is the location of atoms in residue *i* after aligned on the reference, and the angle brackets (< >) are the average of the square distance.

**Fig. 9.**
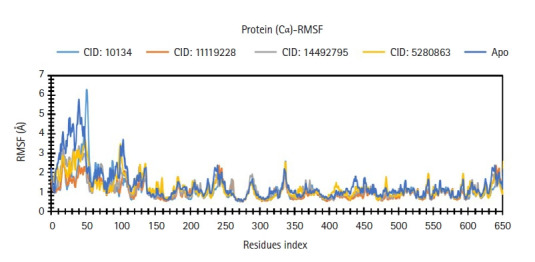
Root-mean-square fluctuation (RMSF) values retrieved from protein residues Cα atoms of the complex structure, viz. CID: 10134 (sky blue), CID: 11119228 (orange), CID: 14492795 (gray) and CID: 5280863 (yellow), and grouper heat shock cognate protein 70 backbone or apo (deep blue) for a 50 ns simulation time.

The significant peaks of variations for CID: 10134 were discovered among 30 to 650 residues maximum, with a fluctuation of 6.3Ȧ, according to the RMSF graph (Fig. 9). The compound also showed a second round of maximum fluctuations with a range of about 5.8Ȧ of apo protein. CID: 5280863 were discovered among 30 to 650 residues maximum, with a fluctuation of 4.2Ȧ. The remaining compounds were discovered to be quite stable, with variations of less than 3.8Ȧ. Nevertheless, as compared to apo, the fluctuations of the compound CID: 5280863 were always acceptable.

## Discussion

Viral encephalopathy and retinopathy, also known as VNN, is a devastating disease that affects a variety of farmed and wild fish species, causing significant losses due to vacuolating lesions of the retina and central nervous system [44]. The virus's genome is made up of two single-stranded positive-sense RNA molecules that are bi-segmented [7]. This virus, which has been found in at least 120 cultured or wild marine and freshwater species, has already wreaked havoc on the aquaculture sector in recent decades, and we may expect it to worsen as a result of global warming [45]. There are currently no specific drugs or vaccines available to prevent or treat infections caused by this deadly disease [45,46]. It has been discovered that the NNV utilizes GHSC70 to enter the host cell, and that inhibiting the virus's entry by targeting the protein which can lower the economic losses cause by the virus [47,48].

According to several studies, neem (*Azadirachta indica*) is the most beneficial traditional medicinal plant on the planet. From antiquity, almost all components of the plant have medicinal characteristics and have been utilized as traditional medicine or cures for a variety of diseases. It is now regarded as a valuable source of unique natural compounds for the creation of medications to treat a variety of illness [49-51]. Furthermore, phytochemicals found in plants may boost the innate immune system, have antibacterial properties, and are redox active molecules with antioxidant properties, all of which may aid in improving the fish's overall physiological state. Many research have looked into the benefits of phytochemicals in disease prevention [52].

CADD is one of most promising tool for selection of novel compounds against a specific protein as its includes of different advance features and techniques [53]. The CADD approaches has minimized the required time and costs involved in entire drug discovery process that make the virtual screening process includes molecular docking, molecular dynamic simulation, and ADMET etc. as integral parts of drug designing [42].

The 3D structure prediction, the identified models were refined and selected the best model (based on the lowest energy score). In the validation test of 3D structure, we found a good number of Z-score (–11.24) and the superior features of most favored, accepted, and disallowed regions for the Ramachandran plot.

In this study, we identified potential drugs by molecular docking and other process. Initially, molecular docking process has used to screen the compounds, where the top 4 compounds has been selected with the highest binding affinities of –9.7 to –9.0 kcal/mol have been chosen for further validation. The RO5 demonstrated the drug like properties of the for the selected compounds [54,55]. All the four compounds were found to follow the five Lipinski’s rules of drug likeness properties. The compound with good ADME properties has been further evaluated through the toxicity properties to measure the harmful effect on humans or animals [56]. Analysis of toxicity found no or less toxicity of the selected four compounds.

The compounds were investigated and optimized by a computational DFT based QM simulation. We retrieved and re-docked the geometry optimized by DFT with the desired protein, and the docking energy was significantly above >9.00 kcal/mol. To determine the reactivity of the compounds, the HOMO-LUMO energy gap was calculated using a FMO model. The HOMO-LUMO gap energy found for all the four compounds were high >3.50 eV which confirms the low reactivity correspondence to the bioactivity of the compound.

Molecular dynamics simulation is used to confirm the stability of a protein in complex with ligands [42,56]. Also, it can determine the stability and rigidity of protein-ligand complexes at a specific artificial environment like body [42]. The RMSD values of the complex systems indicate the best stability of the compounds and RMSF values measures mean fluctuation that determine the compactness of the protein-ligand complex [57]. Therefore, in this study after the molecular dynamic simulation all the four compound PubChem CID: 14492795, 10134, 5280863, and 11119228 showed stabilities against the GHSC70 protein. So, we can conclude that these compounds can be a potential inhibitor against the NNV in fish.

To the best of our knowledge, this study offers the first compressive *in-silico* approaches to identify potential natural antiviral drug candidates against NNV to target GHSC70 protein. An integrative molecular modelling, virtual screening, molecular docking, ADMET, and MDS approaches revealed CID: 11119228 and CID: 14492795 as potential drug candidates that will help to inhibit the activity of the GHSC70 protein of the virus. Further evaluation through different lab-based experiment techniques can help to determine the activity of the compound that will provide alternatives for NNV immunotherapy.

## Figures and Tables

**Table 1.** Validation of selected protein model by Ramachandran and z-score studies

Parameter	Initial model	Refine model	Remarks
Ramachandran			
Highly preferred	0.96649	0.98765	Significant
Preferred	0.02822	0.01058	Significant
Questionable	0.00529	0.00176	Significant
ProSA Web			
Z-score	‒11.01	‒11.24	Significant

**Table 2.** List of absorption, distribution, metabolism, and excretion (ADME) and toxicity of compounds

Property	CID: 14492795	CID: 10134	CID: 11119228	CID: 5280863
Physiochemical properties				
MW (g/mol)	422.51	400.68	274.35	286.24
Heavy atoms	31	29	20	21
Aro. atoms	12	0	6	16
Rotable bonds	6	5	0	1
H-bond acceptors	5	1	3	6
H-bond donors	2	1	2	4
TPSA (Å^2^)	75.99	20.23	57.53	111.13
Lipophilicity				
Log Po/w (Cons)	5.11	6.88	3.25	1.58
Water solubility				
Log S (ESOL)	Soluble	Moderately soluble	Moderately soluble	Soluble
Pharmacokinetics				
GI absorption	High	Low	High	High
BBB permeant	No	No	Yes	No
P-GP substrate	No	No	Yes	No
Drug likeness				
Lipinski violations	0	1	0	0
Medi. chemistry				
Synth. accessibility	Very easy	Easy	Medium	Easy

MW, molecular weight; Aro., aromatic; TPSA, topological polar surface area; GI, gastrointestinal; BBB, BOILED-egg; P-GP, P-glycoprotein.

**Table 3.** The toxicity endpoints of chosen four chemicals include acute toxicity, hepatotoxicity, cytotoxicity, carcinogenicity, and mutagenicity

Classification	Target	CID: 14492795	CID: 10134	CID: 11119228	CID: 5280863
Oral toxicity	LD50 (mg/kg)	2,000	890	760	1190
	Toxicity class	4	4	4	4
Organ toxicity	Hepatotoxicity	Inactive	Inactive	Inactive	Inactive
Toxicity	Carcinogenicity	Inactive	Inactive	Inactive	Inactive
endpoints	Mutagenicity	Inactive	Inactive	Inactive	Inactive
	Cytotoxicity	Inactive	Inactive	Inactive	Inactive

**Table 4.** List of bonding interactions between four phytochemicals and the GHSC70 protein

Ligand ID	Distance	Category	Types	Binding site
CID: 14492795	2.25	Hydrogen bond	Conventional hydrogen bond	ASN454
	3.64	Hydrophobic	Pi-Alkyl	VAL82
	4.76	Hydrophobic	Pi-Alkyl	VAL82
	3.95	Hydrophobic	Pi-Alkyl	HIS227
	4.90	Hydrophobic	Alkyl	PHE150
	5.45	Hydrophobic	Alkyl	LEU399
CID: 10134	2.8368	Hydrogen bond	Conventional hydrogen bond	THR13
	4.83	Hydrophobic	Pi-Alkyl	ARG76
	5.11	Hydrophobic	Pi-Alkyl	ARG76
	4.32	Hydrophobic	Alkyl	ARG72
	4.56	Hydrophobic	Alkyl	PHE150
CID: 11119228	2.15	Hydrogen bond	Conventional hydrogen bond	GLY437
	5.05	Hydrophobic	Pi-Alkyl	ALA406
	5.35	Hydrophobic	Pi-Alkyl	ALA406
	5.47	Hydrophobic	Alkyl	LEU439
CID: 5280863	2.89	Hydrogen bond	Conventional hydrogen bond	THR226
	3.12	Hydrogen bond	Carbon hydrogen bond	THR216
	4.46	Hydrophobic	Pi-Alkyl	ARG72
	5.16	Hydrophobic	Pi-Alkyl	VAL82
	4.47	Electrostatic	Pi-Cation	ASP80
	4.86	Hydrophobic	Pi-Pi T-shaped	PHE150
	4.01	Electrostatic	Pi-Anion	ARG76

GHSC70, grouper heat shock cognate protein 70.

## Supplementary Materials

Supplementary Fig. 1. Ramachandran plot server results of Raptorx predicted protein 3D model.

Supplementary Fig. 2. Z score of predicted 3D model.

Supplementary Fig. 3. Ramachandran plot server result of Galaxy refine protein model.

Supplementary Fig. 4. Z score results of Galaxy refine.

Supplementary Fig. 5. List of compounds and docking score.

Supplementary Fig. 6. (A) Bond length (Angstroms and Bohr) of optimized compound CID 14492795. (B) Bond length (Angstroms and Bohr) of optimized compound CID 10134. (C) Bond length (Angstroms and Bohr) of optimized compound CID 5280863. (D) Bond length (Angstroms and Bohr) of optimized compound CID 11119228.
