# Supplementary figures and images for: Computer-aided drug design of Azadirachta indica compounds against nervous necrosis virus by targeting grouper heat shock cognate protein 70 (GHSC70): quantum mechanics calculations and molecular dynamic simulation approaches
Source: Genomics Inform. 2022 Sep 6;20(3):e33. doi: 10.5808/gi.21063 (PMC9576468; doi:10.5808/gi.21063)

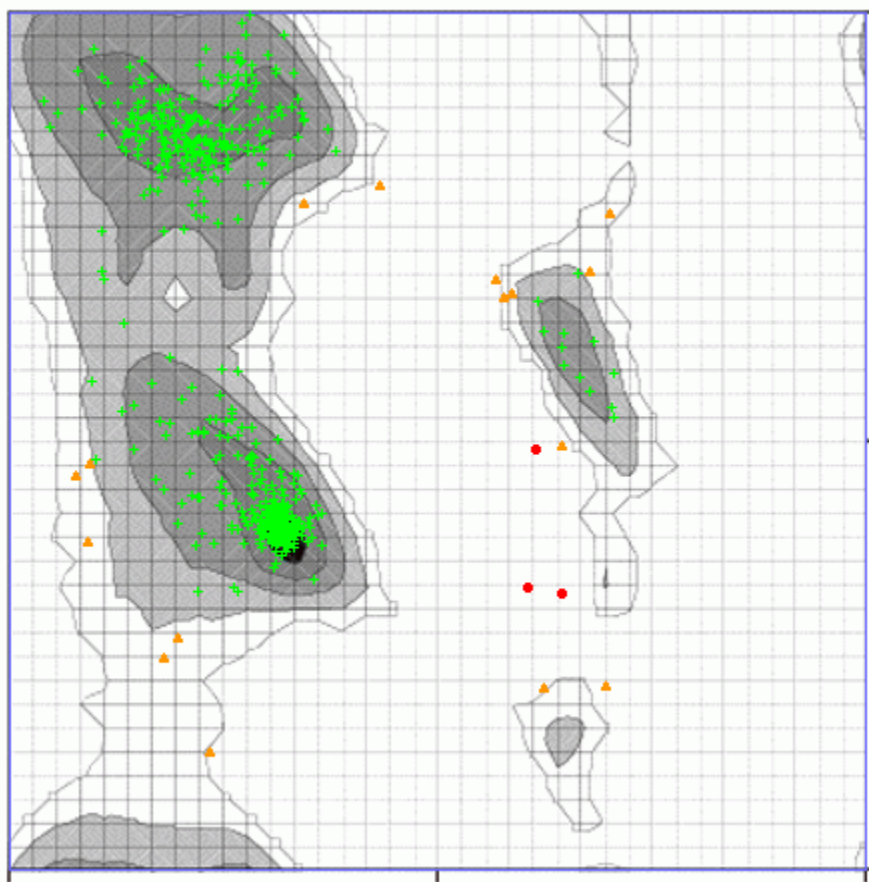

**Supplementary Fig. 1.** Ramachandran plot server results of Raptorx predicted protein 3D model.

Supplement: Supplementary Fig. 1. — Ramachandran plot server results of Raptorx predicted protein 3D model. [file gi-21063suppl1.pdf]

## Overall model quality

Z-Score: **-11.01**

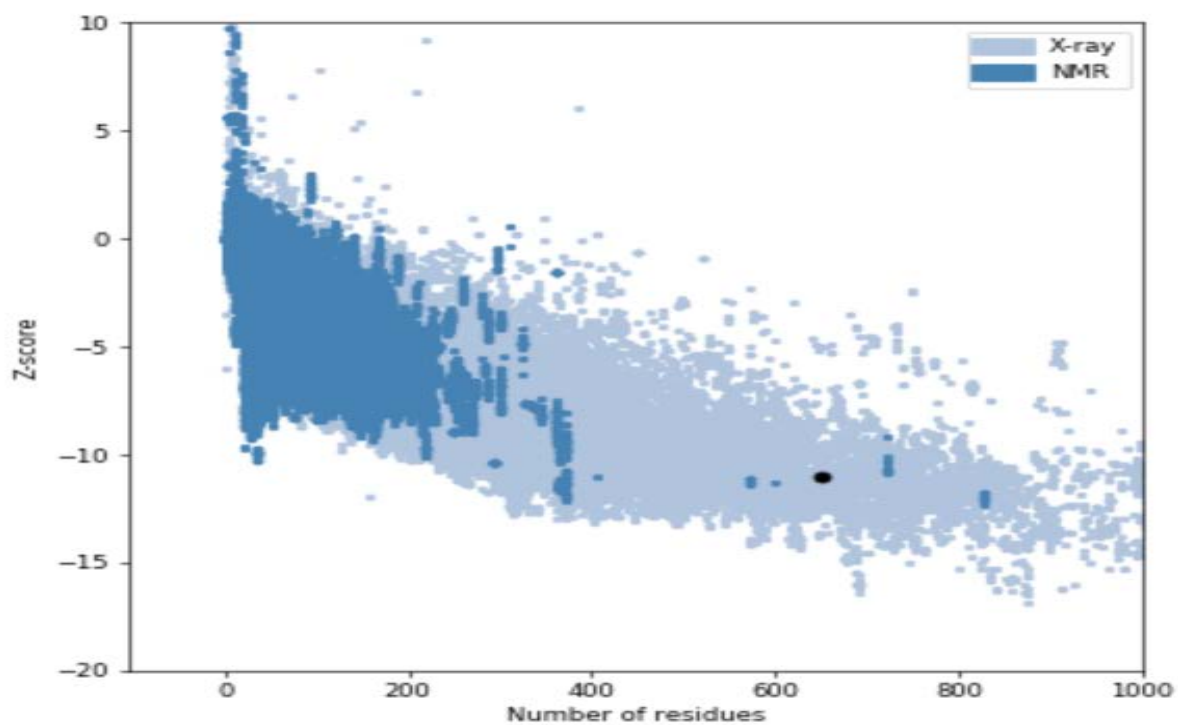

**Supplementary Fig. 2.** Z score of predicted 3D model.

Supplement: Supplementary Fig. 2. — Z score of predicted 3D model. [file gi-21063suppl2.pdf]

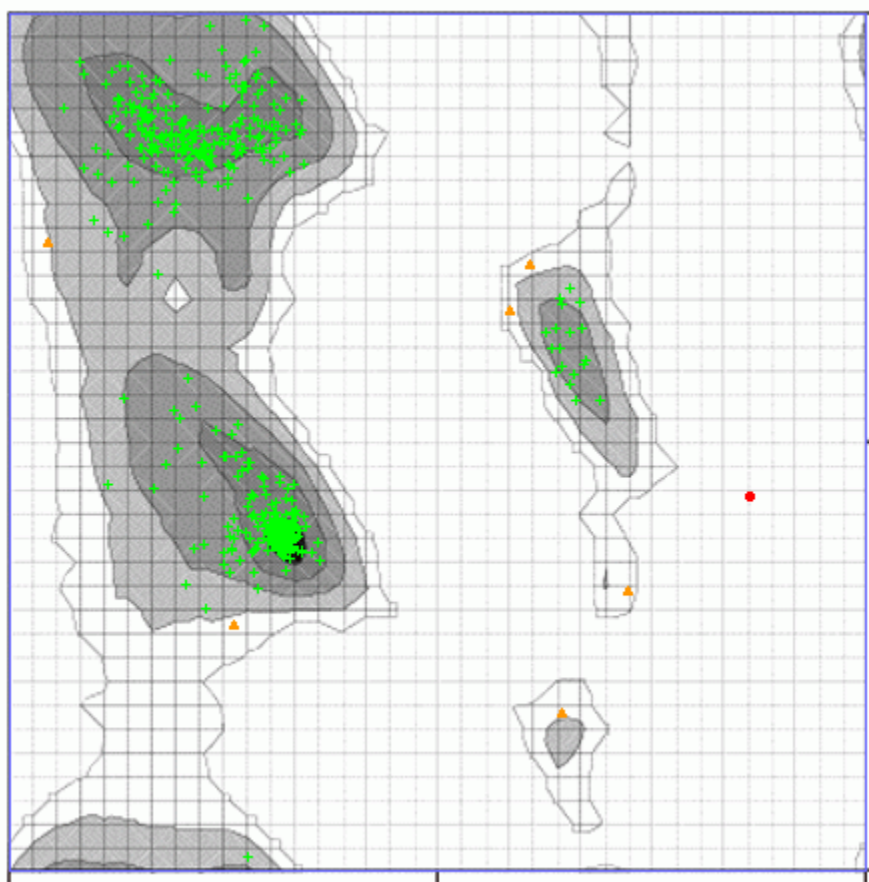

**Supplementary Fig. 3.** Ramachandran plot server result of Galaxy refine protein model.

Supplement: Supplementary Fig. 3. — Ramachandran plot server result of Galaxy refine protein model. [file gi-21063suppl3.pdf]

### Overall model quality

Z-Score: **-11.24**

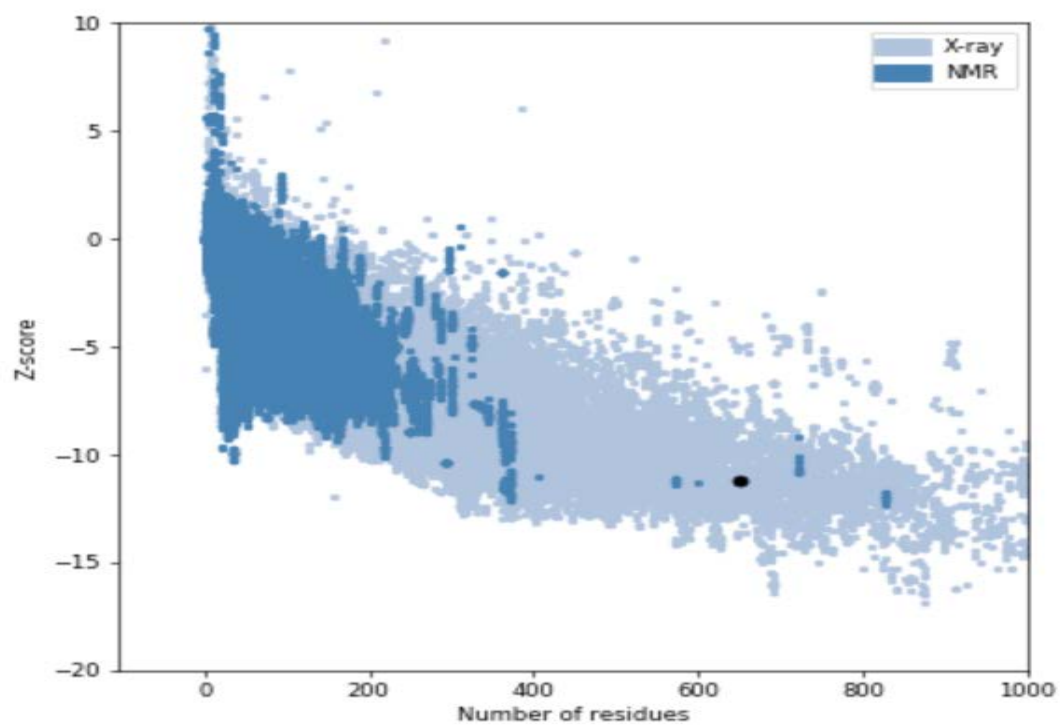

**Supplementary Fig. 4.** Z score results of Galaxy refine.

Supplement: Supplementary Fig. 4. — Z score results of Galaxy refine. [file gi-21063suppl4.pdf]
