# Supplementary material for: Computer-aided drug design of Azadirachta indica compounds against nervous necrosis virus by targeting grouper heat shock cognate protein 70 (GHSC70): quantum mechanics calculations and molecular dynamic simulation approaches
Source: Genomics Inform. 2022 Sep 6;20(3):e33. doi: 10.5808/gi.21063 (PMC9576468; doi:10.5808/gi.21063)
Supplement: Supplementary Fig. 5. — List of compounds and docking score. [file gi-21063suppl5.pdf]

Compound name	Phytochemical name	Docking score	Structure
CID:6442906	Nimocinolide	–8.3	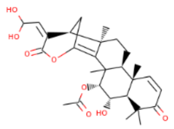
CASID:29803858	((2aR)-8t-[3]furyl-3t,5t-dihydroxy-2a,5a,6a,7-tetramethyl-(2ar,5ac,6ac,9at,10ac,10bc,10ct)-Δ6b-dodecahydro-cyclopenta[d']naphtho[1,8-bc:2,3-b']difuran-6c-yl)-acetic acid	–8.7	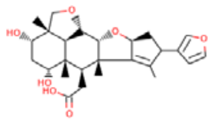
CHEMSPIDER:156225	(5alpha,7alpha,8eta,13alpha,17alpha)-17-(3-Furyl)-4,4,8-trimethyl-3,16-dioxoandrosta-1,14-dien-7-yl acetate	–8.1	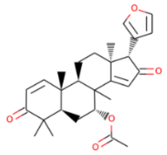
CID:6450192	3-Deacetylsalannin	–8.9	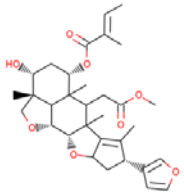
CID:16219576	3beta-Hydroxy-20(29)-lupene	–7.1	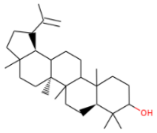
CID:102285347	6-Deacetylnimbinene	–8.1	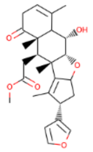
CID:102146586	Azadirachtanin	–8.2	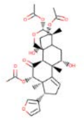

CID:102285346	Desacetylnimbinolide	–7.0	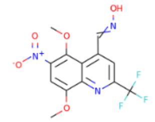
CID:122801	Epoxyazadiradione	–8.3	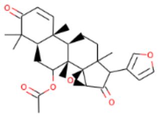
CID:114923	Gedunin	–8.5	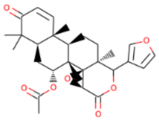
CASID:105377740	Isomargosinolide	–8.0	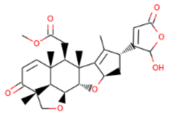
CASID:106807345	Isonimolicinolide	–7.3	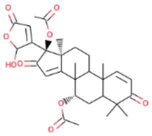
CASID:105404759	Margosinolide	–8.1	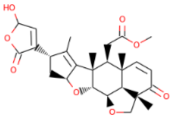
CID:101289833	Meldenin	–6.9	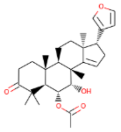
CID:101650342	Melianin B	–7.9	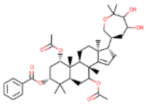

CID:101306757	Nimbidinin	–8.5	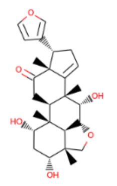
CID:44715635	Nimbinene	–8.3	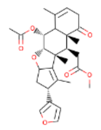
CID:13875774	Nimbocinolide	–8.2	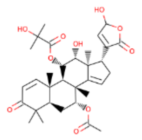
CID:101650373	Nimbolin A	–7.6	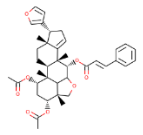
CASID:104522761	Nimocin	–6.9	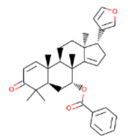
CID:12303662	Phytosterols	–5.9	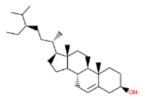
CID:118701505	Salannin	–8.8	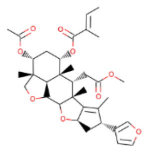
CID:14194026	Salannol acetate	–8.3	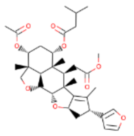
CID:102090424	Vilasinin	–7.1	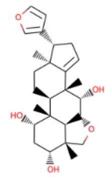

CID:225689	Beta-Amyrin	–4.9	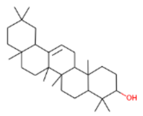
CID:8067	1-Pentanethiol	–5.1	
CID:52952013	1,3-Diacetylvilasinin	–7.0	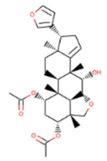
CHEMSPIDER:298060	17-(3-Furyl)-4,4,8-trimethyl-3,16-dioxo-1,2:14,15-diepoxyandrostan-7-yl acetate	–6.9	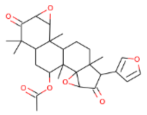
CID:52951894	17-epi-17-Hydroxyazadiradione	–8.9	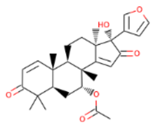
CID:12308716	17-Epiazadiradione	–8.0	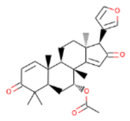
CID:52951892	17-Hydroxyazadiradione	–8.1	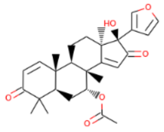
CID:91886694	2',3'-Dehydrosalannol	–7.7	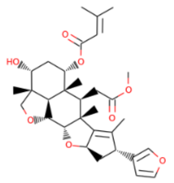

CID:14635659	24-Methylenecycloartan-3-one	–6.6	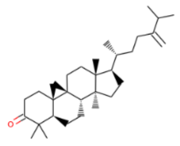
CID:9547213	24-Methylenecycloartanol	–7.1	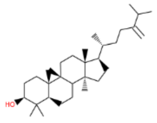
CID:52952216	6-Acetylnimbandiol	–8.8	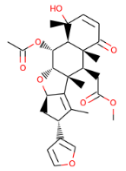
CID:9823926	6beta-Hydroxystigmast-4-en-3-one	–6.1	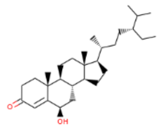
CID:52952112	7-Deacetyl-7-benzoylgedunin	–7.9	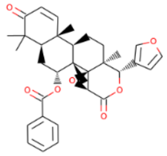
CID:1886	7-Deacetyl-7-oxogedunin	–7.2	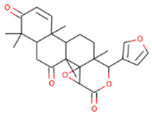
CID:10134	AC1L1UKB	–9.5	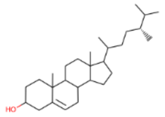
CID:10467	Arachidic acid	–3.9	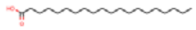

CID:2263	Azadirachtin	–7.9	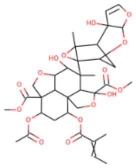
CID:16126804	Azadirachtin B	–8.5	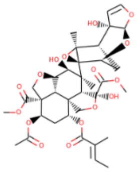
CID:23256847	Azadirachtol	–8.4	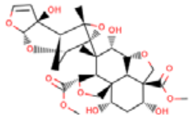
CID:12308714	Azadiradione	–7.9	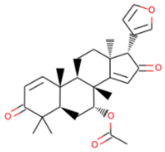
CID:10906239	Azadirone	–6.9	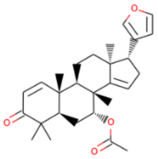
CID:244	Benzyl alcohol	–5.2	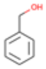
CID:5280489	Beta-carotene	–4.8	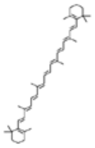

CID:76419085	Bis (acetic acid); tannins	–8.8	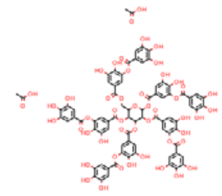
CID:11988279	Campest-4-en-3-one	–6.9	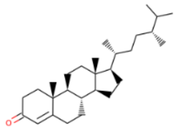
CID:444539	Cinnamic acid	–5.9	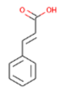
CID:101690	Cycloeucalenol	–6.2	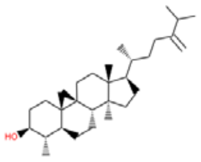
CID:21594790	Cycloeucalenone	–7.3	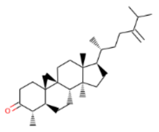
CID:10505484	Deacetylnimbin	–8.9	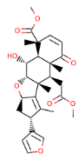
CID:14458886	Deacetylsalannin	–8.0	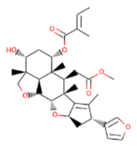
CID:8215	Docosanoic acid	–3.9	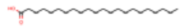

CID:5281416	Esculetin	–6.8	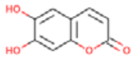
CID:124039	Fraxinellone	–8.3	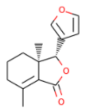
CID:101425842	Isoazadirolide	–8.7	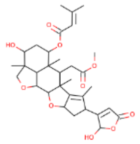
CID:76316558	Isomeldenin	–7.9	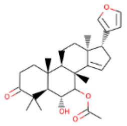
CID:184310	Isonimocinolide	–8.9	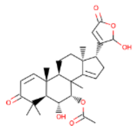
CID:10813969	Isoquercitin	–8.8	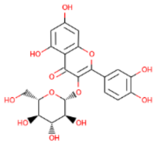
CID:24796982	Isovepaol	–8.9	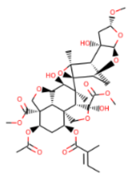
CID:5280863	Kaempferol	–9.1	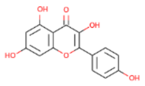

CID:15560423	Kulactone	–8.5	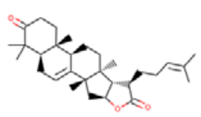
CID:44567124	Kulinone	–8.0	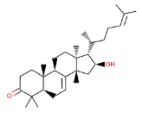
CID:101277363	Melianin A	–8.9	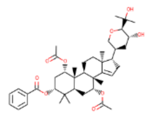
CID:44575793	Melianone	–8.1	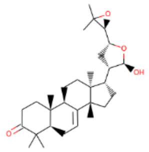
CASID:78916537	Methyl 2-(2-(furan-3-yl)-5,6-dihydroxy-1,6,9a,10a-tetramethyl-9-oxo-3,3a,4a,5,5a,6,9,9a,10,10a-decahydro-2H-cyclopenta[b]naphtho[2,3-d]furan-10-yl)acetate	–8.3	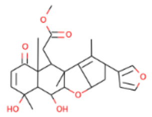
CID:14492795	Nimbaflavone	–9.7	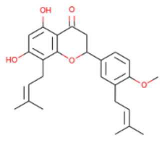
CID:11119228	Nimbidiol	–9.0	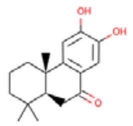

Supplementary Fig. 5. List of compounds and docking score.
