# Supplementary material for: Computer-aided drug design of Azadirachta indica compounds against nervous necrosis virus by targeting grouper heat shock cognate protein 70 (GHSC70): quantum mechanics calculations and molecular dynamic simulation approaches
Source: Genomics Inform. 2022 Sep 6;20(3):e33. doi: 10.5808/gi.21063 (PMC9576468; doi:10.5808/gi.21063)
Supplement: Supplementary Fig. 6. — (A) Bond length (Angstroms and Bohr) of optimized compound CID 14492795. (B) Bond length (Angstroms and Bohr) of optimized compound CID 10134. (C) Bond length (Angstroms and Bohr) of optimized compound CID 5280863. (D) Bond length (Angstroms and Bohr) of optimized compound CID 11119228. [file gi-21063suppl6.pdf]

8	-2.737271874	-1.566446784	-0.712349219
8	-4.001585488	-8.468718201	-3.850424416
8	-5.082096793	-6.278025003	-8.151455884
8	-5.202914534	2.686920125	-8.208107979
8	3.843651638	-1.357382619	9.257128891
6	-1.080413039	-3.628982545	-0.063088502
6	-2.582554338	-6.088065967	-0.236684401
6	0.171817666	-3.063068193	2.449602666
6	-3.536652423	-1.664754110	-3.170151406
6	-3.903486032	-4.028969507	-4.370038592
6	-3.521430680	-6.384116109	-2.926960679
6	-4.028393141	0.624442945	-4.407502409
6	-0.870194251	-3.776619278	4.777361470
6	-4.685770230	-4.088792563	-6.914845653
6	0.337816866	-3.224961019	7.079062842
6	2.452439145	-1.712053951	2.450024075
6	-3.860022334	3.139539735	-3.032626597
6	-4.782694276	0.481462498	-6.958549346
6	-5.094616228	-1.833308219	-8.214850522
6	2.657468745	-1.932813631	7.014175320
6	3.682242583	-1.138164955	4.707554992
6	-0.821460108	-4.063611964	9.565749490
6	-1.217369041	3.956029863	-2.322070728
6	0.229014003	-6.552074955	10.491660423
6	-0.322852126	4.496485827	-0.002859155
6	1.525553114	-7.013532813	12.631756208
6	2.340357607	5.459826562	0.356413660
6	-1.811918411	4.245086130	2.414463211

6	6.258413426	-2.523685594	9.557162576
6	2.419860269	-9.653035934	13.255507513
6	2.213050546	-5.046813719	14.575800480
1	0.440258912	-3.703207203	-1.491039158
1	-1.406840527	-7.727826560	0.260322983
1	-4.256496295	-6.048833365	1.002121692
1	-2.674926034	-4.778196729	4.832337378
1	3.270286097	-1.061067915	0.668326162
1	-4.832031243	-7.642514881	-6.908932701
1	-5.128147525	3.072915555	-1.383761304
1	-4.649393005	4.630140477	-4.252337009
1	-5.644450901	2.313145552	-9.939627998
1	-5.685257644	-1.914900918	-10.190500461
1	6.174392429	-4.560761077	9.133873403
1	7.701000010	-1.607191166	8.363751481
1	6.822477737	-2.289382248	11.544009142
1	-2.878585583	-4.285977911	9.295410960
1	-0.614552120	-2.554571276	10.977633696
1	5.430513132	-0.043845422	4.668350736
1	0.046628989	4.217184326	-3.943725857
1	-0.161577241	-8.149870404	9.230247538
1	3.431099888	4.172609469	1.584826259
1	2.318863863	7.335033786	1.271418983
1	3.360713265	5.645325844	-1.446997205
1	-2.304494387	6.129401833	3.164168534
1	-0.676665523	3.266691838	3.861698521
1	-3.565587908	3.159410204	2.190368165
1	4.504596531	-9.730913432	13.274302728

1	1.747556233	-10.230625013	15.143196377
1	1.740953531	-11.059241513	11.882366470
1	4.237839031	-5.182855093	15.053037550
1	1.132973878	-5.357401524	16.333224862
1	1.863067624	-3.103062354	13.940319645

(A) Bond length (Angstroms and Bohr) of optimized compound CID 14492795.

8	-6.327310000	-2.727140000	2.215810000
6	0.645370000	-0.866990000	0.495850000
6	-0.036600000	-1.313460000	-0.818910000
6	-1.575960000	-1.379690000	-0.800440000
6	-2.190750000	-0.456310000	0.291020000
6	2.141230000	-0.901540000	0.025920000
6	0.103850000	0.536290000	0.867030000
6	-3.731960000	-0.253540000	0.063170000
6	0.771380000	-2.520320000	-1.275810000
6	-1.339920000	0.821370000	0.396170000
6	2.206860000	-2.201030000	-0.829160000
6	-2.196530000	-2.774460000	-0.732390000
6	3.268240000	-0.827890000	1.097660000
6	0.444150000	-1.856950000	1.670330000
6	-4.383520000	-1.618410000	-0.227960000
6	-4.394980000	0.355070000	1.346090000
6	-3.687560000	-2.721150000	-0.567830000
6	-4.001420000	0.710570000	-1.113350000
6	-5.866010000	-1.703310000	0.063730000
6	-5.004410000	-0.706570000	2.264240000
6	3.261820000	0.473540000	1.933100000

6	-6.134380000	-1.471990000	1.560850000
6	4.655240000	-1.038430000	0.469160000
6	3.625820000	1.745780000	1.150840000
6	3.107080000	3.043200000	1.813260000
6	3.315250000	4.257480000	0.861280000
6	3.717500000	3.248300000	3.203610000
6	2.355430000	5.402450000	1.201320000
6	4.753940000	4.779280000	0.826620000
1	-6.451980000	-2.547500000	3.162920000
1	-7.075700000	-0.918700000	1.660500000
1	0.186810000	-0.524660000	-1.559720000
1	2.292280000	-0.071150000	-0.677750000
1	0.137540000	0.677190000	1.953780000
1	0.743480000	1.318050000	0.441700000
1	-0.600320000	-2.104540000	1.857290000
1	0.955540000	-2.810530000	1.503850000
1	0.842940000	-1.441890000	2.602590000
1	-1.875700000	-0.979720000	-1.780400000
1	0.709800000	-2.666290000	-2.358950000
1	0.439070000	-3.446900000	-0.796410000
1	-2.115550000	-0.960010000	1.261170000
1	-1.780170000	-3.348030000	0.102900000
1	-1.965980000	-3.323110000	-1.653260000
1	-1.786850000	1.553730000	1.075620000
1	-1.291140000	1.309610000	-0.585810000
1	2.859410000	-2.054310000	-1.697170000
1	2.606360000	-3.046500000	-0.257480000
1	3.124110000	-1.654770000	1.804890000

1	-5.193990000	1.055070000	1.066390000
1	-3.675690000	0.944940000	1.922960000
1	-5.075470000	0.833460000	-1.295060000
1	-3.602220000	1.709930000	-0.908440000
1	-3.561540000	0.357500000	-2.050730000
1	-4.208540000	-3.669710000	-0.685230000
1	2.290960000	0.588520000	2.420360000
1	3.982410000	0.358810000	2.754010000
1	4.831290000	-0.345030000	-0.359580000
1	4.765210000	-2.056810000	0.083390000
1	5.446630000	-0.891200000	1.212170000
1	-6.378690000	-0.932380000	-0.523810000
1	-6.284210000	-2.665260000	-0.256310000
1	-5.400800000	-0.236310000	3.172400000
1	-4.226160000	-1.399790000	2.609870000
1	3.208900000	1.691990000	0.140600000
1	4.715210000	1.793270000	1.034780000
1	2.023920000	2.920610000	1.949100000
1	3.060080000	3.936180000	-0.157640000
1	4.811760000	3.232500000	3.169500000
1	3.402310000	4.200020000	3.641560000
1	3.397650000	2.458450000	3.891030000
1	2.459040000	6.218940000	0.478290000
1	2.542830000	5.811370000	2.198440000
1	1.315850000	5.059430000	1.162030000
1	4.864260000	5.540280000	0.046070000
1	5.466270000	3.978610000	0.607540000
1	5.039020000	5.243370000	1.776610000

(B) Bond length (Angstroms and Bohr) of optimized compound CID 10134.

8	-0.543231971	0.994884041	0.065444990
8	1.526993085	-5.488635435	-0.857701273
8	-7.901053962	-3.960446153	-1.678084237
8	-3.354658582	-5.946122979	-1.505559813
8	-8.518901764	4.911282571	-0.159216973
8	11.249984785	1.787045837	1.572628078
6	-4.165419062	-1.570247023	-0.814940553
6	-3.072558397	0.797770503	-0.263507171
6	1.081471286	-1.015629453	-0.107136125
6	-2.573997659	-3.756994474	-1.022379554
6	0.122255823	-3.371696352	-0.640779627
6	3.723349792	-0.300131951	0.326979288
6	-6.835915578	-1.740127720	-1.155903813
6	-4.528471329	2.981513289	-0.040876663
6	-7.147119433	2.769155331	-0.383466977
6	-8.305283359	0.437019922	-0.936359227
6	4.348130998	2.237641551	0.856146028
6	5.716952128	-2.079482824	0.236918727
6	6.839283070	2.970186271	1.275999679
6	8.205541731	-1.351241009	0.655549725
6	8.794797979	1.181186457	1.179731368
1	-6.523679932	-5.237524866	-1.747267105
1	-3.669800627	4.803076861	0.385475756

1	0.280860525	-6.809637048	-1.238806092
1	2.855525257	3.654299204	0.938800753
1	5.300541558	-4.052122430	-0.166420609
1	-10.348564702	0.293300591	-1.200134740
1	-10.287439625	4.540595811	-0.429619755
1	7.274532318	4.951826642	1.683218622
1	9.737960220	-2.731523327	0.583919661
1	11.396850510	3.576502965	1.910341009

(C) Bond length (Angstroms and Bohr) of optimized compound CID 5280863.

8	0.540879262	6.829471612	1.256843527
8	7.018028472	0.760112043	-6.758206266
8	7.749307076	5.418396209	-5.002894597
6	-1.186403991	0.127027381	1.323751165
6	-0.615394937	-0.552941383	-1.512748331
6	-3.073660107	-1.618820540	2.813168939
6	-0.002628609	-3.409950075	-1.697070314
6	-2.322227576	-4.408199718	2.351355812
6	-1.989713280	-5.097278851	-0.436407651
6	-1.749408165	2.960499536	1.598989756
6	1.668826469	1.056496667	-2.345594037
6	-2.790943871	0.084895940	-3.374708575
6	-2.780047711	-1.098081977	5.670086924
6	-5.882271027	-1.209570142	2.138926044
6	2.012374874	3.533883975	-1.400903008
6	0.299689755	4.661572289	0.519852281
6	3.388830498	0.171307440	-4.159285011
6	4.034032083	5.038789942	-2.261317928

6	5.389967171	1.663724209	-5.017033527
6	5.714992483	4.133265374	-4.045056744
1	0.645675907	-0.192680241	2.280094245
1	-3.535063164	3.476319142	0.648960250
1	-1.982432166	3.525520047	3.584740280
1	0.214240125	-3.961498730	-3.693412753
1	1.843403135	-3.757850519	-0.785602557
1	-4.490451932	-1.062553239	-3.050539310
1	-2.153620554	-0.278496478	-5.323089769
1	-3.346528981	2.086503156	-3.283436700
1	-3.737575649	-5.650292044	3.252044572
1	-0.516515025	-4.776533770	3.338495756
1	-3.874932272	-2.492382283	6.766906564
1	-0.789365002	-1.246376335	6.270078705
1	-3.472617168	0.788282189	6.211143820
1	-6.462209036	0.770979857	2.414776906
1	-6.347606603	-1.732302365	0.184429698
1	-7.077040835	-2.376336100	3.386714004
1	-3.811312757	-4.956590641	-1.443733648
1	-1.404159006	-7.092013134	-0.604459093
1	3.227640650	-1.712499926	-4.974856733
1	4.223274913	6.942382740	-1.474649565
1	8.253741083	2.066729063	-7.115132042
1	7.782511451	7.122898815	-4.349990489

(D) Bond length (Angstroms and Bohr) of optimized compound CID 11119228.

Supplementary Fig. 6. (A) Bond length (Angstroms and Bohr) of optimized compound CID 14492795. (B) Bond length (Angstroms and Bohr) of optimized compound CID 10134. (C)

Bond length (Angstroms and Bohr) of optimized compound CID 5280863. (D) Bond length (Angstroms and Bohr) of optimized compound CID 11119228.
